# Library of Selenocyanate and Diselenide Derivatives as In Vivo Antichagasic Compounds Targeting *Trypanosoma cruzi* Mitochondrion

**DOI:** 10.3390/ph14050419

**Published:** 2021-05-01

**Authors:** Rubén Martín-Escolano, Daniel Molina-Carreño, Daniel Plano, Socorro Espuelas, María J. Rosales, Esther Moreno, Carlos Aydillo, Carmen Sanmartín, Manuel Sánchez-Moreno, Clotilde Marín

**Affiliations:** 1 Laboratory of Molecular & Evolutionary Parasitology, RAPID Group, School of Biosciences, University of Kent, Canterbury CT2 7NJ, UK; 2 Department of Parasitology, Instituto de Investigación Biosanitaria (ibs.Granada), Hospitales Universitarios de Granada/University of Granada, Severo Ochoa s/n, 18071 Granada, Spain; danidmc94@gmail.com (D.M.-C.); mjrosale@ugr.es (M.J.R.); msanchem@ugr.es (M.S.-M.); 3 Facultad de Farmacia y Nutrición, Departamento de Tecnología y Química Farmacéuticas, Universidad de Navarra, Irunlarrea, E-31008 Pamplona, Spain; dplano@unav.es (D.P.); sespuelas@unav.es (S.E.); emorenoa@unav.es (E.M.); caydillo@unav.es (C.A.); sanmartin@unav.es (C.S.); 4 Instituto de Salud Tropical, Universidad de Navarra, ISTUN, Irunlarrea, E-31008 Pamplona, Spain; 5 Instituto de Investigaciones Sanitarias de Navarra (IdiSNA) Irunlarrea, E-31008 Pamplona, Spain

**Keywords:** American trypanosomiasis, chagas disease, chemotherapy, drug discovery, neglected tropical diseases, screening cascade, selenium derivatives, target product profile, *Trypanosoma cruzi*

## Abstract

Chagas disease is usually caused by tropical infection with the insect-transmitted protozoan *Trypanosoma cruzi*. Currently, Chagas disease is a major public health concern worldwide due to globalization, and there are no treatments neither vaccines because of the long-term nature of the disease and its complex pathology. Current treatments are limited to two obsolete drugs, benznidazole and nifurtimox, which lead to serious drawbacks. Taking into account the urgent need for strict research efforts to find new therapies, here, we describe the in vitro and in vivo trypanocidal activity of a library of selected forty-eight selenocyanate and diselenide derivatives that exhibited leishmanicidal properties. The inclusion of selenium, an essential trace element, was due to the well-known extensive pharmacological activities for selenium compounds including parasitic diseases as *T. cruzi*. Here we present compound **8** as a potential compound that exhibits a better profile than benznidazole both in vitro and in vivo. It shows a fast-acting behaviour that could be attributed to its mode of action: it acts in a mitochondrion-dependent manner, causing cell death by bioenergetic collapse. This finding provides a step forward for the development of a new antichagasic agent.

## 1. Introduction

Chagas disease (CD), also known as American trypanosomiasis, is caused by tropical infection with the insect-transmitted protozoan parasite *Trypanosoma cruzi*. CD is an important public health problem in Latin America since it is a life-long and life-threatening infection, being the major cause of morbimortality in many endemic regions: a total of 6 to 7 million people are infected, and the disease causes about 14 thousand deaths annually. Hematophagous triatomines (vectors) are the principal route of transmission, although congenital route, transmission route from donors to transplant or transfusion recipients, and oral route involving parasite-contaminated food and drink are also important [1,2,3,4].

*T. cruzi* infection is far from innocuous and, in mammal hosts, it is an obligate intracellular parasite for its replication, which can infect most nucleated cells [5,6]. During the initial acute stage of the disease, which occurs 2–8 weeks post-infection in humans, parasite become widely disseminated in tissues and organs and can be detected in the bloodstream, and CD generally manifests as a mild febrile illness. Following suppression of the acute stage by the adaptive immune response [7,8], the disease progresses to a long-lasting asymptomatic chronic stag, which is characterized by an extremely low parasite burden. However, ~30% of those infected will advance to a symptomatic stage, developing pathology such as cardiomyopathy and digestive tract megasyndromes, outcomes for which there are few therapeutic options [9,10,11].

Due to the long-term nature of CD and its complex pathology, the development of vaccines against *T. cruzi* is a challenging goal. Recombinant protein-, DNA-, and viral vector-based vaccines have recently been shown to be protective in mouse models. However, new challenges have to be addressed, such as better endpoints, safety issues and trial design [12,13,14]. The nitroheterocyclic drugs benznidazole (BZN) and nifurtimox (NFX) are the front-line chemotherapy used to treat *T. cruzi* infections for more than 50 years, although treatment failures are frequently reported, in addition to a range of toxic side-effects and extended treatment length [14,15,16,17,18,19]. Furthermore, the well-known cross-resistance, since both drugs require to be activated within the parasite by the same mitochondrial type 1 nitroreductase (TcNTR-1) [20,21], and the natural variation in susceptibility to drugs due to the extreme diversity of species [22], along with the World Health Organization (WHO) lists CD as one of the most neglected tropical diseases, make crucial the international effort aimed at developing new drugs against Chagas disease.

Taking into account the urgent need for new drugs with improved efficacy, tolerability and safety, here, we describe the in vitro and in vivo trypanocidal activity of a library of selected forty-eight selenocyanate and diselenide derivatives [23,24,25] that exhibited leishmanicidal properties. These chemical moieties have been decorated with various substituted aromatic and heteroaromatic rings. Therefore, we explored the importance of the sulfonamide group [23], considering the interest of this motif as linker in described compounds for treatment of trypanosomatid infections [26]. The inclusion of selenium, an essential trace element, was due to the well-known extensive pharmacological activities for selenium compounds including parasitic diseases as *T. cruzi* [27]. On the other hand, it has been demonstrated the interference of selenium in redox processes of the parasite. Some selenium compounds have demonstrated proved efficacy as trypanothione reductase inhibitors the major reductase of trypanosomatids that contributes to redox homeostasis suggesting that its mode of action involves interference with the intracellular redox balance. In addition, most selenoproteins are redox enzymes containing a catalytic selenocysteine residue. The named “TriTryps” selenoproteome consists of a trypanosomatid specific selenoprotein (Seltryp) and two selenoproteins present in other lineages: SelT and SelK. Seltryp possesses two rhodanese domains and an N-terminal selenocysteine containing redox motif [28,29].

## 2. Results and Discussion

### 2.1. In Vitro Biological Evaluation

Initially, the in vitro screening for the forty-eight derivatives was performed on epimastigote form using benznidazole (BZN) as reference drug (Table S1). Twenty-nine of the tested compounds displayed similar effectiveness than the reference with IC_50_ ranged from 0.9 to 18.4 µM. In parallel, and considering the importance of the selectivity index (SI), their cytotoxicity in Vero cells was determined. The SI for the drugs was determined by the ratio of the IC_50_ of Vero cells over the IC_50_ obtained for the corresponding form of the parasite. The cytotoxicity values registered against Vero indicated that the activity of many of these derivatives was not specific against *T. cruzi* as it is showed in the SI. In this context, and with the most exigent criteria established by the literature [15,30,31,32,33], we considered for further progression the compounds that complied with IC_50_ ≤ 10 µM, SI ≥ 10 and 4-fold more selective than the reference (compounds 8, 10, 11, 15, 20, 21, 43, 46, and 47).

Taking into account that the parasite forms relevant to human infection are trypomastigotes and intracellular amastigotes, the derivatives that exhibited the better profiles were tested in these forms (Table 1). This approach is in accordance with the Drugs for Neglected Diseases Initiative guidelines (DNDi) [30]. All of them were more active than BZN in both forms with IC_50_ values between 0.5 and 4.2 µM in amastigotes and 0.9 and 4.5 µM in trypomastigotes. However, it was important to ensure the potential suitability of the compounds analyzing the SI. Good results were found for the hit compounds in all parasite forms tested with SI values exceeding the SI of BZN. The most highlighted were **8** and **10** which contained the diselenide moiety. Particularly **10** presented a SI over 15, 11, and 11 times its registered by the reference for epimastigote, amastigote and BTs, respectively. Derivative **15**, with the selenocyanate entity, showed SI over 4, 4, and 2 times respect to control. We report herein three potential candidates with biological activity against these three trypanosomatids forms that deserved further in vivo investigations.

**Figure 1:**
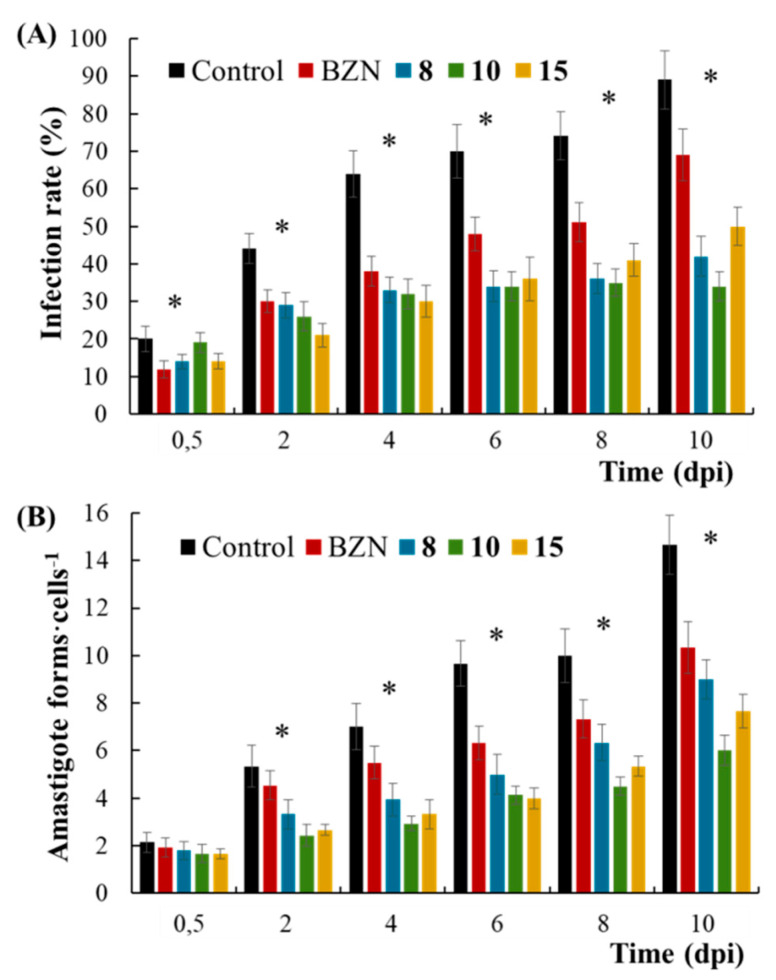
(**A**) Percentage of infected Vero cells and (**B**) number of amastigotes of *Trypanosoma cruzi* per Vero cell exposed to benznidazole (BZN), **8**, **10** and **15**. Values constitute means of three separate determinations ± standard deviation. * Significant differences between untreated and treated parasites for α = 0.05. dpi, days post-infection.

Before going ahead on in vivo trials, the in vitro infection rate and the average number of amastigotes per Vero cell were also measured at 2 days intervals for 10 days after treating the infected cultures with BZN and potential candidates (**8**, **10,** and **15**) (Figure 1) at ICH_25_ concentrations resulting from amastigotes screening. In short, potential candidates produced a decrease in both infection rate and number of amastigotes per cell in a time-dependent manner, showing activity from the first 48 h of treatment. All these potential candidates can be considered as fast-acting compounds since they showed activity from the beginning of the treatment, such as BZN (a drug considered to be fast-acting) [34]. Fast-acting drugs are urgently needed, and this feature is an additional advantage since they can eliminate the parasite in a few doses [35].

### 2.2. In Vivo Biological Evaluation

**Scheme 1:**
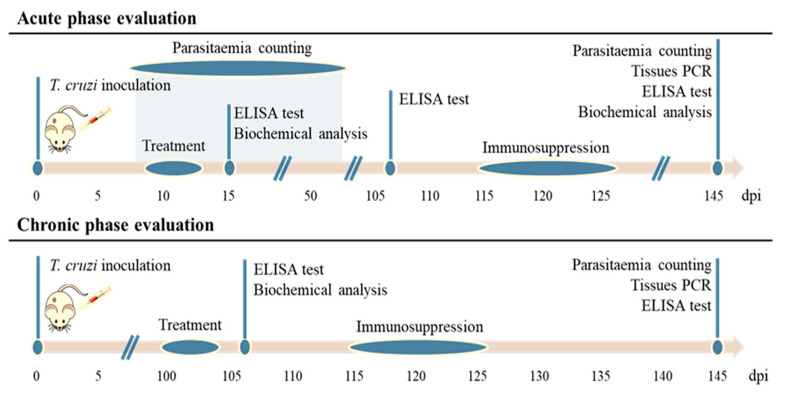
Timeline for all in vivo assays on BALB/c mice for the evaluation of compounds in the acute and chronic phases of Chagas disease. dpi = day post-infection.

Currently, clinical drugs present variable activity in the acute and chronic phases of CD, and the effectiveness of current treatments—especially during chronic CD—is not as effective as they should be [15]. For this reason, and based on the previous in vitro assays, compounds **8**, **10,** and **15** were tested to determine their effectiveness in both phases according to the timeline stablished in the Scheme 1. It should be noted that most in vivo trials have focused on acute CD because of the simplicity of monitoring parasitic load [36]; however, the ability to cure chronic CD is the major demand from a clinical viewpoint [37].

Alternatively, mice were treated once daily for 5 consecutive days—it is defined that a compound showing a reasonable parasitemia reduction following 5 days of treatment can be considered a lead compound [15]—at a subcurative dose of BZN of 20 mg·Kg^−1^·day^−1^—so that the trials could demonstrate whether the potential candidates are more effective (or not) than the reference drug.

**Figure 2:**
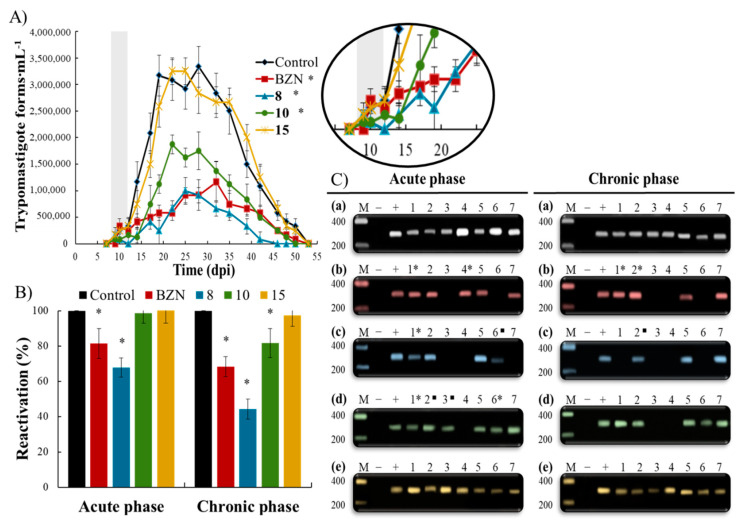
(**A**) Parasitemia profiles of each group of mice infected with *Trypanosoma cruzi* and treated during the acute Chagas disease over a period of 55 days: control (untreated), benznidazole (BZN), **8**, **10** and **15**. Treatment days are represented in grey. Values are the means of three mice ± standard deviation. * Significant differences tween untreated and treated mice for α = 0.05. (**B**) Parasitemia reactivation by fresh blood in the chronic Chagas disease after the immunosuppression cycles for each group of mice treated during the acute and chronic phases of the disease: control (untreated), benznidazole (BZN), **8**, **10,** and **15**. Values are the means of three mice ± standard deviation. * Significant differences between untreated and treated mice for α = 0.05. (**C**) PCR analysis of seven organs/tissues with the *Trypanosoma cruzi* Spliced Leader (SL) intergenic region sequence in the chronic Chagas disease for each group of mice treated during the acute and chronic phases of the disease: (**a**) control (untreated), (**b**) benznidazole, (**c**) **8**, (**d**) **10,** and (**e**) **15**. Lanes: (M) base pair marker, (−) PCR negative control, (+) PCR positive control, (1–7) organs/tissues PCR: (1) adipose, (2) bone marrow, (3) brain, (4) esophagus, (5) heart, (6) lung, (7) muscle. ^■^ 1/3 of the corresponding organ/tissue PCR products showed 300 bp band on electrophoresis; * 2/3 of the corresponding organ/tissue PCR products showed 300 bp band on electrophoresis.

Firstly, the parasitemia levels were determined by counting BTs during the acute CD (Figure 2A). Significant differences were observed between untreated (control) and BZN-treated mice, which showed a remarkable parasitemia reduction even at a subcurative dose. Interestingly, the trypanocidal activity was even stronger for compound **8**; its effect was evident from the beginning of the treatment, showing an undetectable parasitemia on the 12th dpi, being the lowest subsequently, and eliminating or depleting it to undetectable levels on the 46th dpi (that is, 7 days before the parasitemia of untreated and BZN-treated mice). Long-term treatment could be carried out in order to reach higher efficiency. A slight activity was observed for compound **10**, and non-significant differences were observed between untreated and compound **15**-treated mice.

Secondly, the experimental cure was determined using a double checking widely used in in vivo trypanocidal trials. This double checking—based on immunosuppression and PCR (Figure 2B,C, respectively)—was performed to evaluate the treatment effectiveness and the disease extent in both acute and chronic CD at the end of the in vivo timeline. The mice whose parasitemia and PCR remain negative after immunosuppression are considered cured [38,39,40]. 

Immunosuppression enhances the reactivation of any residual infection, which is proportional to the parasite survival rate [41]. Figure 2B shows the reactivation of infection for each mice group compared to untreated (control) mice. As expected, BZN exhibited higher efficacy in chronic CD than in acute CD (reactivations of 81% and 68% in acute and chronic CD, respectively); it is widely known that BZN is more effective in chronic than in acute CD in mice models [38,42], probably because the parasitic load is low and limited to a few locations. Interestingly, compound **8** showed higher efficacy than BZN in both acute and chronic CD, with reactivations of 67% and 44%, respectively. Once again, a light activity was observed for compound **10**, and non-significant differences were observed between untreated and compound **15**-treated mice.

To assess the efficacy of potential candidates by confirming the absence of infection in the target locations, PCR was carried out at the end of the in vivo timeline (Figure 2C). PCR was positive in seven organs/tissues for the untreated (control) mice groups in both phases, showing the presence of the typical nested amastigotes of the chronic CD. BZN-treated mice exhibited 2/7 and 3/7 negative PCR (parasite-free locations) in acute and chronic CD, respectively. Consistent with previous parasitemia and reactivation results, compound **8**-treated mice showed the highest number of negative PCR after treatment in both acute and chronic CD (3/7 and 4/7 parasite-free sites, respectively).

At observed, compound **8** exhibited higher efficacy than BZN at the tested doses in both acute and chronic CD after the double checking of experimental cure, confirming it as a potential candidate for further preclinical research.

Otherwise, the immune status of the mice was evaluated—through the titer of total anti-*T. cruzi* IgG and the splenomegaly (Figure S1)—since it is a characteristic linked to the parasite load and the effectiveness ascribed to the potential candidates [40,43]. In summary, BZN- and compound **8**-treated mice showed significant reduction in both the IgG levels and the splenomegaly, excluding the IgG levels of chronic CD (its samples coming from mice that have undergone a natural history of acute untreated CD). It is interesting to note that compound **8**-treated mice showed very low IgG levels from the beginning to the end of the in vivo timeline in the acute treatment, becoming similar to the cut off value (uninfected mice). Overall, these results confirm the previous results, and demonstrate the in vivo trypanocidal activity of compound **8**. Slight or non-significant reductions were observed for compound **10**- and compound **15**-treated mice in most cases. It has to be mentioned that the samples obtained after IS do not reflect data indicating infection rates, but confirm the IS suffered by the mice.

Finally, biochemical analyses were performed to assess the toxicity associated to the treatment of compound **8**. Kidney, heart and liver biochemical markers were measured, including values from uninfected and BZN-treated mice (Table S2). Most of the biochemical markers were disturbed 2 days after treatment for both BZN and compound **8**, but returned to near-normal levels over time. Notably, none of the mice died nor lost more than 10% body mass after treatment. The low toxicity allows compound **8** to be tested at higher doses, establishing an improved treatment schedule based on pharmacokinetics in order to reach a total parasite clearance.

### 2.3. Mode of Action Studies

**Figure 3:**
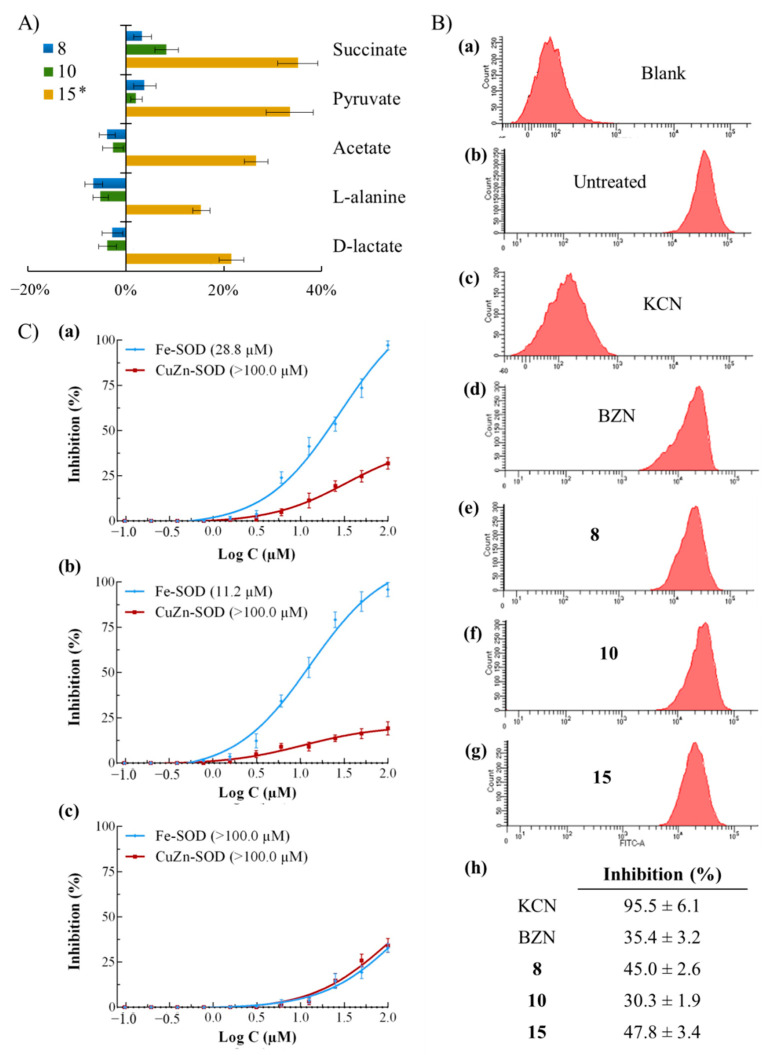
(**A**) Variation among peaks of catabolites excreted by epimastigotes of *Trypanosoma cruzi* exposed to **8**, **10** and **15** at IC_25_ concentrations in comparison to control (untreated) parasites incubated 72 h. Values constitute means of three separate determinations ± standard deviation. * Significant differences between untreated and treated parasites for α = 0.05. (**B**) Mitochondrial membrane potential from epimastigotes of *Trypanosoma cruzi* exposed to benznidazole (BZN) and compounds at their IC_25_ concentrations incubated 72 h: (**a**) blank, (**b**) untreated (control), (**c**) potassium cyanide (KCN), (**d**) BZN, (**e**) **8**, (**f**) **10**, (**g**) **15**. (**h**) Inhibition, in percentage, on mitochondrial membrane potential with respect to untreated parasites. Values constitute means of three separate determinations ± standard deviation. Significant differences between untreated and treated parasites for α = 0.05. (**C**) In vitro inhibition (%) of *Trypanosoma cruzi* Fe-SOD (activity 42.0 ± 3.8 U·mg^−1^) and human erythrocytes CuZn-SOD (activity 47.3 ± 4.1 U·mg^−1^) for (**a**) **8**, (**b**) **10,** and (**c**) **15**. Values constitute means of three separate determinations ± standard deviation. In brackets: IC_50_ value.

The mode of action studies were initiated at the glycolytic and mitochondrial levels, since fast-acting and trypanocidal activities exhibited by the potential candidates could be explained by a bioenergetic collapse. It is widely known that *T. cruzi* catabolizes glucose at a high rate, excreting to the medium incompletely oxidized metabolites such as pyruvate and succinate, among others [44,45]. Hence, these metabolites were qualitatively and quantitatively analyzed by ^1^H RMN and compared with those found for untreated parasites (Figure 3A). As shown, metabolites from compound **8**- and compound **10**-treated parasites were not substantially altered (<10% variation). However, the excretion of all metabolites from compound **15**-treated parasites was strikingly increased concerning the corresponding untreated parasites. Succinate and pyruvate were the most increased excretions, which are likely to be result of a decreased ATP synthesis due to mitochondrial dysfunction. This ATP deficit is balanced by the so-called Crabtree effect, that is, by an increase in the activity of the glycolytic pathway [46]. Alternatively, an increase in succinate could also be a consequence of redox stress produced by Fe-SOD inhibition [47]. Consequently, the hypothetical mitochondrial dysfunction and enzyme Fe-SOD inhibition was evaluated.

Mitochondrial integrity was evaluated by flow cytometry after staining the parasites with Rho (Figure 3B). KCN-treated parasites were included as control samples with fully depolarized mitochondria [48]. It is widely known that BZN causes, among others, respiratory chain inhibition through its transformation to highly reactive metabolites [49], decreasing the mitochondrial membrane potential (35.4%). All tested compounds caused a considerable depolarization in the mitochondrial membrane potential, being similar o even higher than those caused by BZN (from 30.3 to 47.8%). Therefore, the glycolytic alteration observed by compound **8** could be explained by a mitochondrial dysfunction and an ATP deficit. Overall, since the mitochondrion balances the NADH/NAD+ and ATP/ADP ratios and plays a crucial role in cell death decisions [50,51,52], the cidal mode of action of these compounds could be construed as a bioenergetic collapse before cell death in a mitochondrion-dependent manner. This mode of action could be the cause of the fast-acting trypanocidal activities of these compounds.

Alternatively, mitochondrial dysfunctions can cause a reduction in nucleic acid levels [44], which were determined by flow cytometry (Figure S2). Remarkably, all treated parasites showed higher reductions than BZN in nucleic acid levels (from 44.9 to 69.5% for compounds, and 22.4% for BZN). Since the reduction in nucleic acids could also be due to random degradation attributed to necrosis [51], the biological consequences of compounds were more than probably due to a cidal mode of action.

Finally, Fe-SOD enzyme inhibition assays were performed. Fe-SOD is one of the most relevant therapeutic targets for CD because of its structural/biochemical differences with the human CuZn-SOD [53] and its protective effects against oxidative stress [54]. SOD inhibition curves are shown in Figure 3C. Compounds **8** and **10** were selective for the parasitic enzyme Fe-SOD, reaching 100% inhibition at 100 µM. However, their IC_50_ values are too high to attribute trypanocidal activity to the inhibition of this enzyme. Compound **15** showed no inhibitory activity.

## 3. Materials and Methods

### 3.1. Chemistry

**Figure 4:**
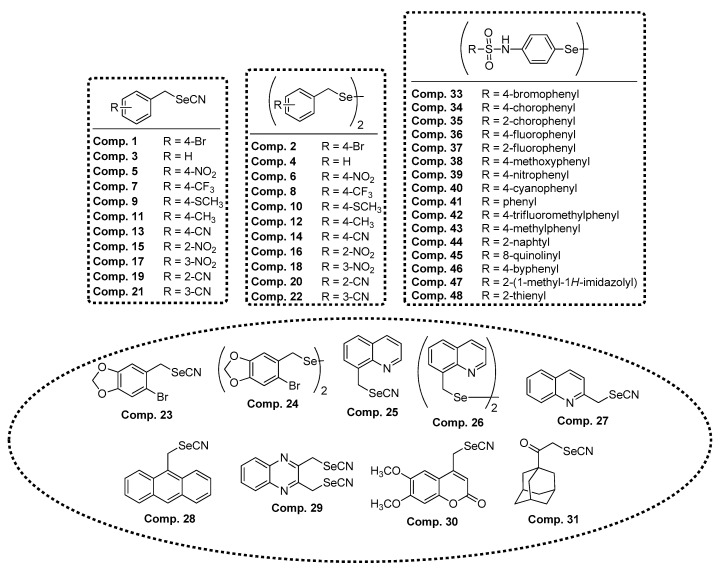
Chemical structures of the Se compounds presented in this work.

The compounds (Figure 4) were prepared using the previously reported procedures. Briefly, selenocyanate derivatives (compounds **1**, **3**, **5**, **7**, **9**, **11**, **13**, **15**, **16**, **17**, **18**, **23**, **24**, **26**, **27**, **29**, **30,** and **31**) were obtained from the corresponding halides by nucleophilic substitution in the presence of potassium selenocyanate (KSeCN) in acetone. The diselenide (compounds 2, **4**, **6**, **8**, **10**, **12**, **14**, **19**, **20**, **21**, **22**, **25**, **28,** and **32**) were prepared by reduction of the corresponding selenocyanates with sodium borohydride in ethanol. Finally, the selenosulfonamides (compounds 33–48) were synthesized by reaction of 4,4’-diaminodiphenyldiselenide with different sulfonyl chlorides in ethyl ether.

### 3.2. In Vitro Activity Assays

#### 3.2.1. Screening against Extracellular Epimastigotes

Trypanocidal activity against epimastigotes of *T. cruzi* Arequipa strain (MHOM/Pe/2011/Arequipa, discrete typing unit V) was tested as previously described [55,56]. In short, 5 × 10^5^ epimastigotes·mL^−1^ were treated by adding the tested compounds at a concentration range from 100 to 0.2 µM in 96-well microplates (200 µL·well^−1^) for 48 h. Subsequently, resazurin sodium salt (Sigma-Aldrich) was added to be incubated for further 24 h. Finally, the absorbance was measured, and the trypanocidal activity was expressed as the IC_50_ (inhibitory concentration 50) using GraphPad Prism 6 software. Each compound concentration was tested in triplicate in three independent experiments.

#### 3.2.2. Cytotoxicity Test

Mammalian Vero cells (EACC No. 84113001), cultured as previously reported [57], were used to determine the cytotoxicity of the tested compounds [56]. In short, 1.25 × 10^4^ Vero cells·mL^−1^ were treated by adding the compounds at a concentration range from 400 to 1 µM in 96-well microplates (200 µL·well^−1^) at 37 °C for 48 h. Subsequently, resazurin sodium salt (Sigma-Aldrich) was added to be incubated for further 24 h. Finally, cell viability was determined following the same procedure as described to assess the trypanocidal activity in the epimastigotes. Each compound concentration was tested in triplicate in three independent experiments.

#### 3.2.3. Screening against Intracellular Amastigotes and Infected Cells

Trypanocidal activity against amastigotes was determined according to the literature reported previously [42]. In short, culture derived trypomastigotes (obtained as previously described [56]) were used to infect 1 × 10^4^ Vero cells·well^−1^ in 24-well microplates with rounded coverslips at a multiplicity of infection (MOI) ratio of 10:1 for 24 h. After that, non-phagocyted trypomastigotes were washed and the plates were treated by adding the tested compounds at a concentration range from 50 to 0.1 µM in 500 mL·well^−1^ at 37 °C in 95% humidified air and 5% CO_2_ atmosphere. After 72 h of incubation, the trypanocidal effect was determined based on the number of amastigotes and infected cells in methanol-fixed and Giemsa stained preparations by analyzing 500 host cells randomly distributed in microscopic fields, and the activity was expressed as the IC_50_ using GraphPad Prism 6 software. Each compound concentration was tested in triplicate in four separate determinations.

#### 3.2.4. Screening against Bloodstream Trypomastigotes

2 × 10^6^ Bloodstream trypomastigotes (BTs), obtained by cardiac puncture from infected BALB/c mice as previously described [56], were treated by adding the tested compounds at a concentration range from 50 to 0.1 µM in 96-well microplates (200 µL·well^−1^) at 37 °C. After 24 h of treatment, resazurin sodium salt (Sigma-Aldrich) was added to be incubated for further 4 h [42]. Finally, trypanocidal activity was determined following the same procedure as described to assess the trypanocidal activity in the epimastigotes. Each compound concentration was tested in triplicate in three independent experiments.

### 3.3. In Vivo Activity Assays on BALB/c Mice

#### 3.3.1. Ethics Statement

All animal work (protocols and procedures, Scheme 1) was performed under RD53/2013 and approved by the Ethics Committee on Animal Experimentation (CEEA) of the University of Granada, Spain. Female BALB/c mice aged 10–12 weeks and ~20 g were maintained under standard conditions.

#### 3.3.2. Infection and Treatment

Infection was carried out by intraperitoneal inoculation of 5 × 10^5^ BTs of *T. cruzi* per mouse in 0.2 mL PBS [56]. The mice were treated by oral route (~200 µL) once daily for 5 consecutive days. The treatment for mice treated in the acute and chronic phases began when the infection was confirmed (9th day post-infection (dpi)) and was established that the animals had entered the chronic phase (100th dpi), respectively [42]. 

The mice were divided into six groups (*n* = 3 per group): 0, negative control group (uninfected and untreated mice); I, positive control group (infected and untreated mice); II, BZN group (mice infected and treated with BZN); III, 8 group (mice infected and treated with 8); IV, 10 group (mice infected and treated with 10); V, 15 group (mice infected and treated with 15). The tested compounds and BZN were prepared at 2 mg·mL^−1^ in an aqueous suspension vehicle containing 5% (*v/v*) DMSO and 0.5% (*w/v*) hydroxypropyl methylcellulose, as previously reported [38]. Therefore, doses of 20 mg·kg^−1^ per day were administered for 5 consecutive days, and vehicle only was administered in the negative and positive control groups.

#### 3.3.3. Screening Assays on Mice

All in vivo assays for the evaluation of compounds in the acute and chronic CD (Scheme 1)—monitoring of parasitemia [56], immunosuppression [58], PCR [59,60], ELISA [56], and clinical analysis [56]—were performed as previously described. PCR was based on the Spliced Leader (SL) intergenic region sequence of *T. cruzi* (for detailed description, see literature reported previously).

### 3.4. Mode of Action Studies

#### 3.4.1. H Nuclear Magnetic Resonance (NMR) Analysis of Excreted Metabolites

5 × 10^5^ epimastigotes·mL^−1^ of *T. cruzi* Arequipa were treated by adding the tested compounds at IC_25_ concentrations in 25 cm^2^ cell culture flasks at 28 °C for 72 h. Untreated controls were also included. Cultures were then centrifuged and filtered, and the metabolites of the supernatants were analyzed using a ^1^H NMR spectrometer (VARIAN DIRECT DRIVE 500 MHz Bruker) with AutoX probe, D_2_O as solvent and 2,2-dimethyl-2-silapentane-5-sulphonate as the reference signal [61]. Chemical shifts were expressed in parts per million (ppm), and analyses were conducted as previously reported [62].

#### 3.4.2. Flow Cytometry Analysis of Mitochondrial Membrane Potential and Nucleic Acid Levels

The untreated and treated epimastigotes of *T. cruzi* described in the NMR analysis were collected by centrifugation, washed three times in PBS and stained with 10 mg·mL^−1^ rhodamine 123 (Rho) (Sigma-Aldrich) or acridine orange (AO) (Sigma-Aldrich) dyes in 0.5 mL PBS for 20 min at 28 °C [63]. Control epimastigotes with a fully depolarized mitochondrion were obtained by incubation for 40 min with 10 mM KCN prior to Rho loading [48]. Non-stained parasites were also included. After elapsed time, epimastigotes were immediately processed and analyzed by flow cytometry as previously reported [62].

#### 3.4.3. SOD Enzymatic Inhibition Analysis

Excreted Fe-SOD protein—obtained and quantified as previously described [64]—and commercial Cu/Zn-SOD from human erythrocytes (Sigma-Aldrich) were exposed to the tested compounds at a concentration range from 100 to 0.1 µM to determine in vitro activities using the method previously described [65].

### 3.5. Statistical Analyses

Statistical analyses were performed by using SPSS software (v. 21, IBM). The *t*-test for paired samples was used to verify whether there were differences between the assays used, with a value of *p* < 0.05 considered statistically significant and with a 95% confidence level. In addition, statistical studies based on contingency tables (prevalence) were conducted.

## 4. Conclusions

A library of forty-eight selenocyanate and diselenide derivatives were screened against *T. cruzi*. We identified compound **8** as a promising alternative that represents a step forward in the development of new drugs against CD in both acute and chronic infection. Compound **8** shows higher trypanocidal activity against the three morphological forms, lower toxicity, and higher efficacy after in vivo treatment than the reference drug BZN. In addition, compound **8** shows a fast-acting behaviour that could be attributed to its mode of action: it acts in a mitochondrion-dependent manner, causing cell death by bioenergetic collapse due to mitochondrial dysfunction. It is worth considering long-term treatment, or even combined therapies, because of its lack of toxicity with the aim of achieving a sterile cure.

## Figures and Tables

**Table 1:** Activity of benznidazole and compounds tested against the three developmental forms of *Trypanosoma cruzi*, toxicity on mammalian Vero cells, and selectivity index.

Comp	Activity IC_50_ (µM) ^a^			Toxicity IC_50_ (µm) ^b^ Vero Cell	Selectivity Index ^c^		
	Epimast.	Amast.	Trypomast.		Epimast.	Amast.	Trypomast.
**BZN**	16.9 ± 1.8	8.3 ± 0.7	12.4 ± 1.1	80.4 ± 7.1	5	10	7
**8**	1.9 ± 0.1	0.8 ± 0.2	1.5 ± 0.3	44.3 ± 2.7	23 (5)	55 (6)	30 (4)
**10**	1.8 ± 0.2	1.2 ± 0.2	1.7 ± 0.3	134.6 ± 6.3	75 (15)	112 (11)	79 (11)
**11**	3.0 ± 0.4	3.1 ± 0.1	4.5 ± 0.6	61.3 ± 3.7	20 (4)	20 (2)	14 (2)
**15**	0.9 ± 0.1	0.5 ± 0.0	1.3 ± 0.3	17.9 ± 1.0	20 (4)	36 (4)	14 (2)
**20**	1.3 ± 0.2	1.0 ± 0.1	0.9 ± 0.0	16.4 ± 0.5	13 (3)	16 (2)	18 (3)
**21**	1.0 ± 0.2	0.8 ± 0.1	0.9 ±0.1	21.7 ± 1.8	22 (4)	27 (3)	24 (3)
**43**	5.1 ± 0.6	4.2 ± 0.7	3.7 ± 0.2	66.9 ± 6.2	13 (3)	16 (2)	18 (3)
**46**	2.7 ± 0.8	2.3 ± 0.1	2.0 ± 0.3	48.6 ± 3.5	18 (4)	21 (2)	24 (3)
**47**	3.6 ± 0.3	3.0 ± 0.4	3.8 ± 0.4	44.7 ± 3.0	12 (2)	15 (2)	12 (2)

^a^ Inhibition concentration 50 (IC_50_), concentration (µM) required to inhibit 50% population, determined using GraphPad Prism 6. ^b^ Towards Vero cells. ^c^ Selectivity index (SI), IC_50_ Vero cells/IC_50_ developmental forms of the parasite. Values are the means of three separate determinations ± standard deviation. In brackets: number of times that compounds exceed the reference drug SI. BZN, benznidazole.

## Data Availability

The data presented in this study are available in this article and Supplementary Material.

## Supplementary Materials

The following are available online at https://www.mdpi.com/article/10.3390/ph14050419/s1, Figure S1: (A) Anti-*Trypanosoma cruzi* immunoglobulin G levels, (B) Weight percentage of spleens in the chronic Chagas disease, Figure S2: Nucleic acids levels of *Trypanosoma cruzi*, Table S1: Activity of benznidazole and compounds tested against cultured epimastigote form of *Trypanosoma cruzi*, toxicity against cultured Vero cells, and selectivity index, Table S2: Clinical analysis.
