# Supplementary material for: Library of Selenocyanate and Diselenide Derivatives as In Vivo Antichagasic Compounds Targeting Trypanosoma cruzi Mitochondrion
Source: Pharmaceuticals (Basel). 2021 May 1;14(5):419. doi: 10.3390/ph14050419 (PMC8147293; doi:10.3390/ph14050419)
Supplement: Supplementary file 1 [file pharmaceuticals-14-00419-s001.zip › pharmaceuticals-1176133-supplementary.pdf]

*Article*

# Library of Selenocyanate and Diselenide Derivatives as In Vivo Antichagasic Compounds Targeting *Trypanosoma Cruzi* Mitochondrion

**Rubén Martín-Escolano [1,*], Daniel Molina-Carreño [2], Daniel Plano [3,4,5], Socorro Espuelas [3,4,5], María J. Rosales [2], Esther Moreno [3,4,5], Carlos Aydillo [3,4,5], Carmen Sanmartín [3,4,5], Manuel Sánchez-Moreno [2] and Clotilde Marín [2,*]**

1 Laboratory of Molecular & Evolutionary Parasitology, RAPID Group, School of Biosciences, University of Kent, Canterbury CT2 7NJ, UK
2 Department of Parasitology, Instituto de Investigación Biosanitaria (ibs.Granada), Hospitales Universitarios De Granada/University of Granada, Severo Ochoa s/n, 18071 Granada, Spain; danidmc94@gmail.com (D.M.-C.); mjrosale@ugr.es (M.J.R.); msanchem@ugr.es (M.S.-M.)
3 Facultad de Farmacia y Nutrición, Departamento de Tecnología y Química Farmacéuticas, Universidad de Navarra, Irunlarrea, E-31008 Pamplona, Spain; dplano@unav.es (D.P.); sespuelas@unav.es (S.E.); emorenoa@unav.es (E.M.); caydillo@unav.es (C.A.); sanmartin@unav.es (C.S.)
4 Instituto de Salud Tropical, Universidad de Navarra, ISTUN, Irunlarrea, E-31008 Pamplona, Spain
5 Instituto de Investigaciones Sanitarias de Navarra (IdiSNA) Irunlarrea, E-31008 Pamplona, Spain
* Correspondence: r.martin-escolano@kent.ac.uk (R.M.-E.); cmaris@ugr.es (C.M.)

*Supplementary material*

**Figure S1.** A) Anti-*Trypanosoma cruzi* immunoglobulin G levels. B) Weight percentage of spleens in the chronic Chagas disease.

**Figure S2.** Nucleic acids levels of *Trypanosoma cruzi*.

**Table S1**. Activity of benznidazole and compounds tested against cultured epimastigote form of *Trypanosoma cruzi*, toxicity against cultured Vero cells, and selectivity index.

**Table S2.** Clinical analysis.

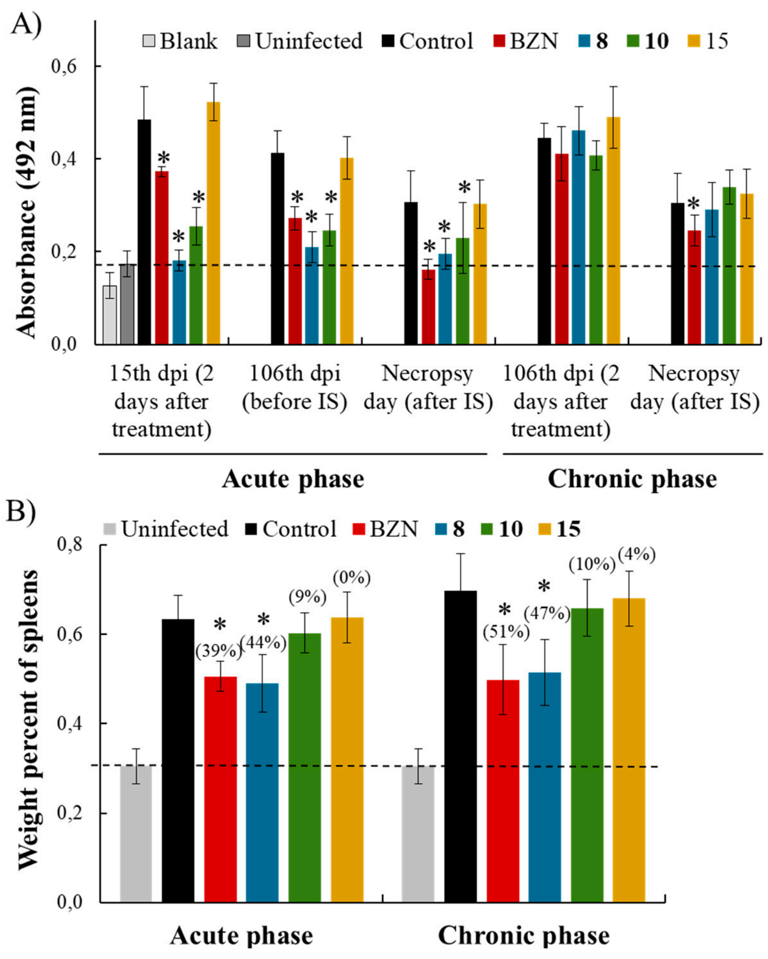


**Figure S1**. **A**) Anti-*Trypanosoma cruzi* immunoglobulin G levels, expressed in absorbance at 492 nm, at different days post-infection (dpi) for each group of mice treated during the acute and chronic phases of Chagas disease: control (untreated), benznidazole (BZN), **8**, **10** and **15**. Blank and uninfected mice are also included. Dashed line shows the cut off for uninfected mice. Values are the means of three mice ± standard deviation. (IS) immunosuppression. * Significant differences between untreated and treated mice for $\alpha$ = 0.05. **B**) Weight percentage of spleens in the chronic Chagas disease for each group of mice treated during the acute and chronic phases of the disease: control (untreated), benznidazole (BZN), **8**, **10** and **15**. Values are the means of three mice ± standard deviation. In brackets: reduction of splenomegaly in comparison to the control. * Significant differences between untreated and treated mice for $\alpha$ = 0.05.

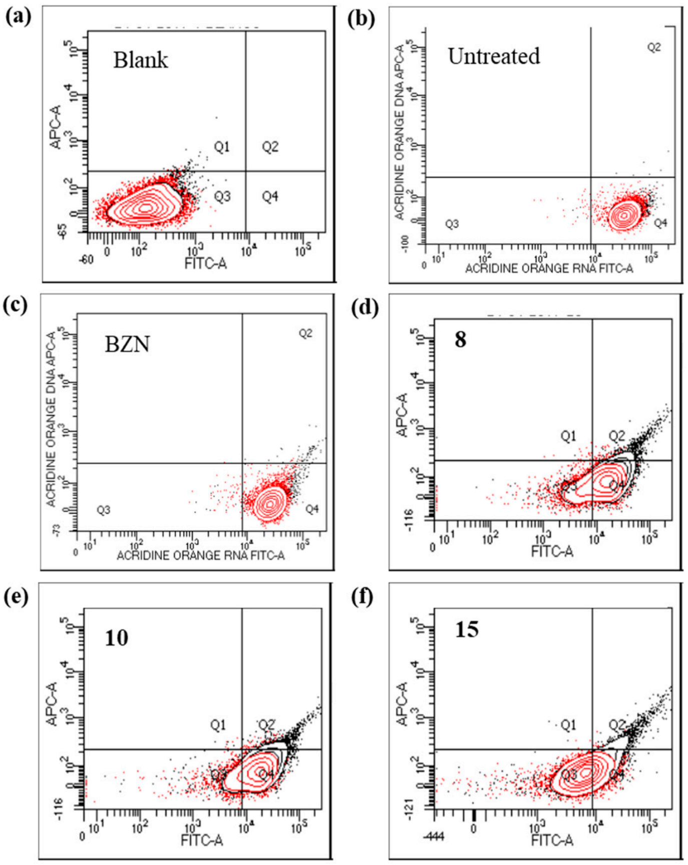


(g)

| | Inhibition (%) | | Inhibition (%) |
|---|---|---|---|
| BZN | 22.4 ± 3.6 | **8** | 51.8 ± 4.4 |
| **10** | 44.9 ± 5.1 | **15** | 69.5 ± 5.8 |

**Figure S2.** Nucleic acids levels of *Trypanosoma cruzi* exposed to benznidazole (BZN) and compounds at their $IC_{25}$ concentrations incubated 72 h: (**a**) blank, (**b**) untreated (control), (**c**) BZN, (**d**) **8**, (**e**) **10**, (**f**) **15**. (**g**) Inhibition, in percentage, in the nucleic acids levels with respect to untntreated parasites. Values constitute means of three separate determinations ± standard deviation. Significant differences between untreated and treated parasites for $\alpha = 0.05$.

**Table S1**. Activity of benznidazole and compounds tested against cultured epimastigote form of *Trypanosoma cruzi*, toxicity against cultured Vero cells, and selectivity index.

| Comp | Activity IC50 (μM)[a] | Toxicity $IC_{50}$ (μM)[b] Vero cell | SI [c] |
|---|---|---|---|
| **BZN** | 16.9 ± 1.8 | 80.4 ± 7.1 | 5 |
| 1 | 39.1 ± 2.5 | 68.4 ± 3.7 | 2 (0) |
| 2 | 39.3 ± 4.1 | 30.7 ± 2.1 | 1 (0) |
| 3 | 34.5 ± 2,6 | 53.3 ± 2.5 | 1 (0) |
| 4 | 35.4 ± 3.6 | 26.8 ± 1.3 | 1 (0) |
| 5 | 21.8 ± 2.2 | 28.6 ± 2.4 | 1 (0) |
| 6 | 13.6 ± 0.7 | 28.2 ± 1.1 | 2 (0) |
| 7 | 34.1 ± 2.6 | 50.5 ± 3.8 | 1 (0) |
| 8 | 1.9 ± 0.1 | 44.3 ± 2.7 | 23 (5) |
| 9 | 11.3 ± 0.7 | 67.3 ± 4.9 | 6 (1) |
| 10 | 1.8 ± 0.2 | 134.6 ± 6.3 | 75 (15) |
| 11 | 3.0 ± 0.4 | 61.3 ± 3.7 | 20 (4) |
| 12 | 19.4 ± 1.5 | 4.9 ± 0.7 | 0 (0) |
| 13 | 1.9 ± 0.7 | 12.8 ± 0.7 | 7 (1) |
| 14 | 27.9 ± 2.9 | 8.9 ± 1.5 | 0 (0) |
| 15 | 0.9 ± 0.1 | 17.9 ± 1.0 | 20 (4) |
| 16 | 18.4 ± 2.3 | 18.9 ± 2.4 | 1 (0) |
| 17 | 37.4 ± 2.6 | 81.2 ± 4.8 | 2 (0) |
| 18 | 1.5 ± 0.3 | 7.3 ± 1.1 | 5 (1) |
| 19 | 29.1 ± 3.0 | 18.5 ± 0.7 | 0 (0) |
| 20 | 1.3 ± 0.2 | 16.4 ± 0.5 | 13 (3) |
| 21 | 1.0 ± 0.2 | 21.7 ± 1.8 | 22 (4) |
| 22 | 39.7 ± 2.6 | 29.8 ± 3.0 | 0 (0) |
| 23 | 14.1 ± 0.9 | 38.9 ± 2.1 | 3 (1) |
| 24 | 45.7 ± 2.6 | 61.8 ± 4.1 | 1 (0) |
| 25 | 10.3 ± 0.8 | 12.6 ± 0.3 | 1 (0) |
| 26 | 6.7 ± 0.4 | 19.6 ± 1.0 | 3 (1) |
| 27 | 99.5 ± 6.1 | 68.3 ± 6.7 | 0 (0) |
| 28 | 21.4 ± 0.9 | 169.2 ± 9.7 | 8 (2) |
| 29 | 32.1 ± 1.1 | 67.4 ± 3.5 | 2 (0) |
| 30 | 30.9 ± 1.4 | 108.6 ± 10.5 | 4 (1) |
| 31 | 13.2 ± 0.8 | 85.5 ± 4.9 | 6 (1) |
| 32 | 7.6 ± 0.7 | 56.5 ± 3.9 | 7 (1) |
| 33 | 10.2 ± 0.8 | 49.7 ± 3.2 | 5 (1) |
| 34 | 41.3 ± 2.1 | 29.8 ± 3.4 | 0 (0) |
| 35 | 28.4 ± 1.7 | 69.0 ± 5.3 | 2 (0) |
| 36 | 11.1 ± 0.8 | 70.8 ± 4.8 | 6 (1) |
| 37 | 46.1 ± 3.1 | 39.6 ± 3.1 | 0 (0) |
| 38 | 27.8 ± 1.6 | 40.3 ± 2.5 | 1 (0) |
| 39 | 17.1 ± 1.3 | 11.7 ± 2.7 | 0 (0) |
| 40 | 3.8 ± 0.1 | 16.0 ± 1.7 | 4 (1) |
| 41 | 6.8 ± 0.7 | 26.5 ± 1.4 | 4 (1) |
| 42 | 5.9 ± 0.4 | 36.0 ± 2.3 | 6 (1) |
| 43 | 5.1 ± 0.6 | 66.9 ± 6.2 | 13 (3) |

| | | | |
|---|---|---|---|
| 44 | 5.6 ± 1.1 | 30.8 ± 2.2 | 6 (1) |
| 45 | 1.7 ± 0.3 | 14.3 ± 1.1 | 8 (2) |
| 46 | 2.7 ± 0.8 | 48.6 ± 3.5 | 18 (4) |
| 47 | 3.6 ± 0.3 | 44.7 ± 3.0 | 12 (2) |
| 48 | 1.8 ± 0.0 | 15.7 ± 1.1 | 9 (2) |

[a] Inhibition concentration 50 ($IC_{50}$), concentration (μM) required to inhibit 50% growth, determined using GraphPad Prism 6. [b] Towards Vero cells. [c] Selectivity index (SI), $IC_{50}$ Vero cells / $IC_{50}$ epimastigote. Data in brackets refer to the number of times that compounds exceed the reference drug SI. Values are the means of three independent experiments ± standard deviation. BZN, benznidazole.

**Table S2.** Clinical analysis determined at different days post-infection in groups of mice infected with *Trypanosoma cruzi* and treated with benznidazole and compounds.

| | **Kidney marker profile** | | **Heart marker profile** | | **Liver marker profile** | | | |
|---|---|---|---|---|---|---|---|---|
| | **Urea (mg/dL)** | **Uric acid (mg/dL)** | **CK-MB (U/L)** | **LDH (U/L)** | **AST/GOT (U/L)** | **ALT/GPT (U/L)** | **Total bilirubin (mg/dL)** | **ALP (U/L)** |
| **Uninfected mice** | 38 [32–40] | 4.4 [4.0 –5.1] | 492 [150–630] | 3121 [2505–3851] | 153 [132–177] | 50 [46–62] | 0.23 [0.22 –0.31] | 165 [141–192] |
| 15th dpi (Control) | 31 | 4.3 | 535 | 3275 | 167 | 60 | 0.23 | 180 |
| 15th dpi and BZN (2 days after treatment) | -- | ---- | = | = | ++++ | ++ | ++++ | = |
| 15th dpi and **8** (2 days after treatment) | ---- | ---- | + | = | = | +++ | --- | ---- |
| Necropsy day of mice (Control) | 34 | 4.0 | 496 | 2761 | 179 | 49 | 0.21 | 161 |
| Necropsy day of mice and BZN | -- | - | - | = | + | = | ++ | = |
| Necropsy day of mice and **8** | - | --- | = | = | + | + | - | -- |

CK-MB, creatine kinase-muscle/brain. LDH, lactate dehydrogenase. AST/GOT, aspartate aminotransferase. ALT/GPT, alanine aminotransferase. ALP, Alkaline phosphatase. BZN, benznidazole. dpi, day post-infection. n= 3, except for uninfected mice (n = 6).

Key: =, variation ≤ 10%; +/-, 10-20% increase/decrease over the range; ++/--, 20-30% increase/decrease over the range; +++/---, 30-40% increase/decrease over the range; ++++/----, > 40% increase/decrease over the range.
